# Glial cell diversity and methamphetamine-induced neuroinflammation in human cerebral organoids

**DOI:** 10.1038/s41380-020-0676-x

**Published:** 2020-02-12

**Authors:** Jason Dang, Shashi Kant Tiwari, Kriti Agrawal, Hui Hui, Yue Qin, Tariq M. Rana

**Affiliations:** 1 Division of Genetics, Department of Pediatrics, Institute for Genomic Medicine, Program in Immunology, University of California San Diego, 9500 Gilman Drive MC 0762, La Jolla, CA 92093 USA; 2 Department of Biology, Bioinformatics Program, University of California San Diego, 9500 Gilman Drive MC 0762, La Jolla, CA 92093 USA

**Keywords:** Stem cells, Diseases

## Abstract

Methamphetamine (METH) is a potent stimulant that induces a euphoric state but also causes cognitive impairment, neurotoxicity and neurodevelopmental deficits. Yet, the molecular mechanisms by which METH causes neurodevelopmental defects have remained elusive. Here we utilized human cerebral organoids and single-cell RNA sequencing (scRNA-seq) to study the effects of prenatal METH exposure on fetal brain development. We analyzed 20,758 cells from eight untreated and six METH-treated cerebral organoids and found that the organoids developed from embryonic stem cells contained a diverse array of glial and neuronal cell types. We further identified transcriptionally distinct populations of astrocytes and oligodendrocytes within cerebral organoids. Treatment of organoids with METH-induced marked changes in transcription in multiple cell types, including astrocytes and neural progenitor cells. METH also elicited novel astrocyte-specific gene expression networks regulating responses to cytokines, and inflammasome. Moreover, upregulation of immediate early genes, complement factors, apoptosis, and immune response genes suggests a neuroinflammatory program induced by METH regulating neural stem cell proliferation, differentiation, and cell death. Finally, we observed marked METH-induced changes in neuroinflammatory and cytokine gene expression at the RNA and protein levels. Our data suggest that human cerebral organoids represent a model system to study drug-induced neuroinflammation at single-cell resolution.

## Introduction

Methamphetamine (METH) is a stimulant which induces a temporary state of intense euphoria; however, studies have shown that METH also commonly leads to psychosis, depression, and attenuated immune systems [1, 2]. In 2005, the economic impact of METH use in the United States alone was $23.4 billion [3] and as many as 1.3 million people over the age of 12 have reported having used METH [1]. Clinical studies have shown that acute and chronic METH abuse induces system-wide alterations to the brain on both the structural and biochemical level [4]. In fact, users of METH may exhibit reduced hippocampal volume which is correlated with poor memory [4, 5]. In addition, other structural alterations in the brain associated with METH abuse include changes in the shape of the corpus collosum and enlargement of the frontal lateral ventricle [4]. In the case of prenatal exposure, studies have shown that fetal exposure to METH can cause in fetal growth restriction [6, 7]; decrease hippocampal, putamen, and globus pallidus volume [4, 8]; and cognitive deficits including impaired attention, memory, and visual motor integration [8]. As such, METH addiction poses a global problem, yet the precise molecular mechanisms by which METH provokes these structural and biochemical changes in the brain have yet to be elucidated.

Because METH affects a wide range of biological processes including oxidative stress, apoptotic cell death, metabolism, dopamine and serotonin dysfunction, and cytokine regulation [1, 9–11], studying the mechanisms by which METH works in 2D homogenous neuronal cultures may be insufficient. Thus, we propose the use of cerebral organoid models, which are composed of neural progenitor cells, glial and neuronal cell types grown in a 3D self-organizing manner, to study the effect of METH on the human brain. Organoid are able to differentiate, self-organize, and form complex, physiologically relevant structures, thus making them ideal in vitro models of human brain development, disease pathogenesis, and drug screening which are dependent on heterogeneous cellular interactions [12–18].

Previous studies have utilized single-cell RNA sequencing (scRNA-seq) to dissect the cellular composition of cerebral organoids; however, these studies have focused on the dynamic nature of neural stem cell (NSC) and neuronal populations within organoid [15, 17, 19]. Glial cells, including astrocytes, are often characterized as merely support cells within the brain. Yet, astrocytes make up the largest population of glial cells and are essential for regulating neurotransmitter recycling, synaptogenesis, and providing trophic and metabolic support for neurons [20]. Moreover, along with microglia and oligodendrocytes, astrocytes modulate the brain’s neuroimmune system in response to infection and drug treatment. Thus, astrocytes play integral functions in brain development, neuronal function, and disease etiology and/or pathology.

METH treatment is known to perturb neurotransmission, neuronal apoptosis, metabolism, and synaptic plasticity, and induce cytokine expression [9, 21]. Glial cells, particularly astrocytes, dynamically interact with neuronal cells to modulate many of these functions that contribute to METH pathogenesis [22]. Yet, the role of astrocytes in METH has not been fully explored [21]. Interestingly, recent studies have indicated that the induction of innate immune genes may contribute to addiction [23]. Thus, further studies into the roles of glial cells, and particularly astrocytes, during METH pathogenesis and addiction may elucidate novel neuronal–glial interactions.

Here we utilized cerebral organoids and scRNA-seq to study the effects of prenatal METH exposure and evaluate glial cell diversity within organoids with single-cell resolution. First, using scRNA-seq we dissected the cellular diversity and observed the dynamic transcriptome during differentiation and self-organization within cerebral organoids. In particular, we analyzed astrocyte and oligodendrocyte subtypes observed within organoids. Second, organoids were treated with METH over time to study the effect of prenatal METH exposure on the brain. scRNA-seq identified differentially expressed genes and novel gene co-expression networks regulating the cortical astrocyte response to cytokines, neuroinflammation, inflammasome activation, and cell cycle regulation induced by METH treatment. In all, we present a platform for studying both the effects of drugs on the human brain as well as human glial cell biology and function with single-cell resolution in vitro.

## Results

### Cellular diversity of human cerebral organoids revealed by scRNA-seq

**Fig. 1:**
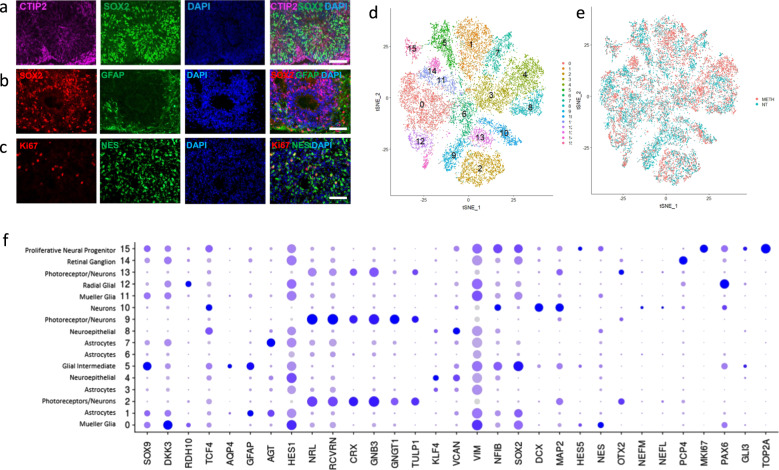
Characterization of human cerebral organoids with single-cell resolution. **a** Cerebral organoids immunostained for cortical neuronal marker CTIP2, neural stem cell marker Nestin, and DAPI show radial cortical layer organization around ventricle-like structures at 20×. Scale bar represents 100 μm. **b** Cerebral organoids immunostained for neural stem cell marker SOX2, astrocyte marker GFAP, and DAPI show glial and neural stem cell populations at 20×. Scale bar represents 100 μm. **c** Cerebral organoids immunostained for proliferation marker Ki67, neural stem cell marker Nestin, and DAPI show the presence of proliferating neural stem cells at 20×. Scale bar represents 100 μm. **d** t-distributed stochastic neighbor embedding (tSNE) plot of scRNA-seq data from eight untreated and six METH-treated cerebral organoids (10,135 cells from control organoids and 10,623 METH-treated cells after quality control filtering) generated by Seurat identified 16 distinct clusters. Cerebral organoids were treated with 5 μM methamphetamine for a week and analyzed by scRNA-seq. **e** t-distributed stochastic neighbor embedding (tSNE) plot of the same data as Fig. 1d, split by treatment, with red representing cells from METH treatment and blue presenting cells from the control. **f** Dot plot of canonical genes to classify tSNE clusters 0–15 (from Fig. 1d). Cluster identities are labeled on the left and canonical marker genes are located on the bottom. Darker shades of blue and size of dots represent greater expression of genes and percentage of cells expressing the gene.

To study the effect of METH on the human brain in vitro, self-organizing human cerebral organoid models were generated using the previously established “intrinsic” method [12, 16, 24]. Embryoid bodies were generated from human embryonic stem cells (hESCs) and differentiated into self-organizing three-dimensional cerebral organoids in bioreactors. Immunostaining showed the radial organization of CTIP2+ cortical neurons and SOX2+ NSCs around ventricle-like structures within the cerebral organoids (Fig. 1a). In addition, immunostaining revealed the presence of a GFAP+ astrocyte population (Fig. 1b) and both Ki67+ proliferative and Ki67− quiescent NSCs (Fig. 1c).

To determine the effect of prenatal METH abuse on the human brain, six 10-month-old cerebral organoids were treated with a 5 μM METH, a physiologically relevant concentration, for a week. To better elucidate the cellular composition of these cerebral organoids, we performed Gel Bead-In-EMulsion (GEM) based scRNA-seq on control and METH-treated cerebral organoids. To characterize the transcriptomes of individual cells, cerebral organoids were enzymatically dissociated and loaded into a microfluidic, droplet-based system to encapsulate cells—along with reverse transcription reagents and a two-dimensional indexing barcode system containing unique molecular identifiers and cell-specific barcodes—for massively parallel transcriptional profiling. Eight control and six METH-treated organoids were dissociated and analyzed by scRNA-seq (10,135 cells from control organoids and 10,623 METH-treated cells after quality control filtering).

To evaluate the cell-type-specific effect of METH on gene expression in the brain, we first aggregated the scRNA-seq datasets from all treated and untreated organoids and clustered cells based on their expression of commonly variable genes following the Seurat integrative analysis pipeline [25]. Analysis by t-distributed stochastic neighbor embedding revealed 16 transcriptionally distinct clusters (Fig. 1d) containing both treated and untreated cells (Fig. 1e) from two batches of organoids. Utilizing known cellular markers (Camp et al. [15]; Quadrato et al. [17], R&D Systems (https://www.rndsystems.com/resources/cell-markers/neural-cells/astrocytes/astrocyte-markers), clusters were classified to reveal the presence of *DKK3*+ *SOX9*+ Müller glia (cluster 0 and 11), *GFAP*+ astrocytes (cluster 1), *NRL*+ *RCVRN*+ *CRX*+ *GBN3*+ *GNGT1*+*, TULIP1*++ neurons (cluster 2, 9, and 13), *HES1*+ *GLUL*+ astrocytes (cluster 3 and 6), *VIM*+ *VCAN*+ *KLF4*+ neuroepithelial cells (cluster 4 and 8), *GFAP*+ *SOX2*+ *NFIB*+ glial intermediate cells (cluster 5), *AGT*+ astrocytes (cluster 7), *MAP2*+ *DCX*+ neurons (cluster 10), *PAX6*+ *SOX2*+ radial glial cells (cluster 12), *PCP4*+ *NEFL*+ *NEFM*+ retinal ganglion cells (cluster 14), *TOP2A*+ *GLI3*+ *PAX6*+ *SOX2*+ proliferative neural progenitor cells (cluster 15), (Fig. 1f). These results illustrate the neural progenitor cells, neurons, and glial cellular diversity within cerebral organoid models.

Based on these clustering analyses, we analyzed both the cellular and transcriptional heterogeneity associated with “intrinsically” differentiated organoids. As expected, we found a difference in cellular compositions between organoids and batches (Fig. S1A–C), consistent with previous publications observing an inherent heterogeneity within cerebral organoid models [12, 17]. However, averaging the transcriptional profiles of cells from individual organoids within each cluster (Fig. S1A–C) suggested that while the cellular compositions of each organoid vary, the transcriptomes are largely conserved across organoids and batches within each identified cluster. Thus, comparing bulk RNA-seq data between individual organoids may be biased due to differing organoid heterogeneity but single-cell approaches can be utilized to determine drug effects within specific cell types with minimal bias induced by organoid heterogeneity.

### METH-induced differential gene expression in single-cell resolution

**Fig. 2:**
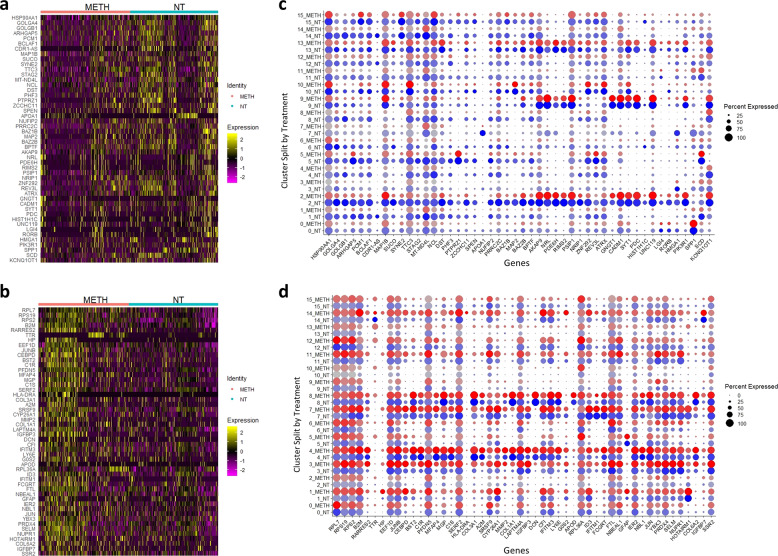
Top genes up and downregulated by METH treatment. **a** A heatmap with 48 downregulated genes as a response to METH treatment. The genes were found using Seurat to identify the markers that differed between the two treatment groups. The METH subset consists of all METH cells from all clusters together, represented as one group. **b** A heatmap with the top 50 upregulated genes identified as a response to METH treatment. The genes were found using Seurat to identify the markers that differed between the two treatment groups. The METH subset consists of all METH cells from all clusters together, represented as one group. **c** A dot plot of the 48 genes downregulated by meth treatment. Each cluster is divided by treatment, where blue dots represent cells from control organoid and red dots represent cells from METH-treated organoids. Darker shades of blue and red and size of dots represent greater expression of genes and percentage of cells expressing the gene. **d** A dot plot of the top 50 genes upregulated by meth treatment. Each cluster is divided by treatment, where blue dots represent cells from control organoid and red dots represent cells from METH-treated organoids. Darker shades of blue and red and size of dots represent greater expression of genes and percentage of cells expressing the gene.

To determine the effect of METH on organoids in a cell-type-specific manner, we performed differential gene expression analysis between control and METH-treated group (Fig. 2 and S2). Heatmap and dot-plot analysis showed the top 48 genes downregulated by METH treatment compared with control organoids in different cell-type-specific clusters (Fig. 2a, b). The genes which downregulated are mostly involved in the regulation of NSC proliferation and neuronal differentiation during brain development. Gene ontology (GO) analysis showed top nine downregulated pathways related to neurogenesis, neuronal system development, and cellular processes (Fig. S2B). In addition, we further identified top 50 genes are upregulated in response to METH treatment (Fig. 2c, d). Further, GO analyses revealed that top 20 upregulated pathways belonged to inflammation/immune function, stimulus, cytokines, and oxidative stress response categories (Fig. S2A). Within the central nervous system, astrocytes play a key role in modulating brain development, neuronal plasticity, and the brain’s response to drug abuse [26, 27]. Indeed, METH treatment induced a robust effect at the transcriptomic level within the GFAP+ cortical astrocyte population in organoids (Fig. 2).

### METH-induced neuroinflammation in cerebral organoid model

**Fig. 3:**
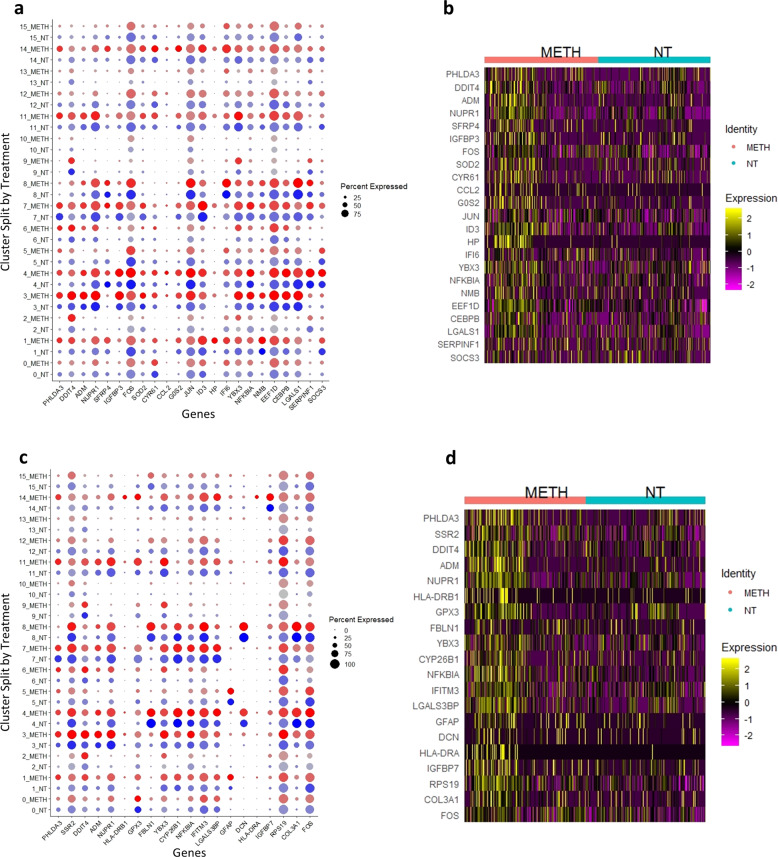
METH treatment upregulates the genes related to inflammation, apoptosis and stress. **a** A dot plot of the genes from the set of genes upregulated by METH (Fig. 2b) treatment identified by PANTHER to be related to apoptosis and cell death. Each cluster is divided by treatment, where blue dots represent cells from control organoid and red dots represent cells from METH-treated organoids. Darker shades of blue and red and size of dots represent greater expression of genes and percentage of cells expressing the gene. **b** A heatmap of the same genes from Fig. 3a separated by treatment, for a holistic view of the data. **c** A dot plot of the genes from the set of genes upregulated by METH (Fig. 2b) treatment identified by PANTHER to be related to stress. Each cluster is divided by treatment, where blue dots represent cells from control organoid and red dots represent cells from METH-treated organoids. Darker shades of blue and red and size of dots represent greater expression of genes and percentage of cells expressing the gene. **d** A heatmap of the same genes from Fig. 3c separated by treatment, for a holistic view of the data.

**Fig. 4:**
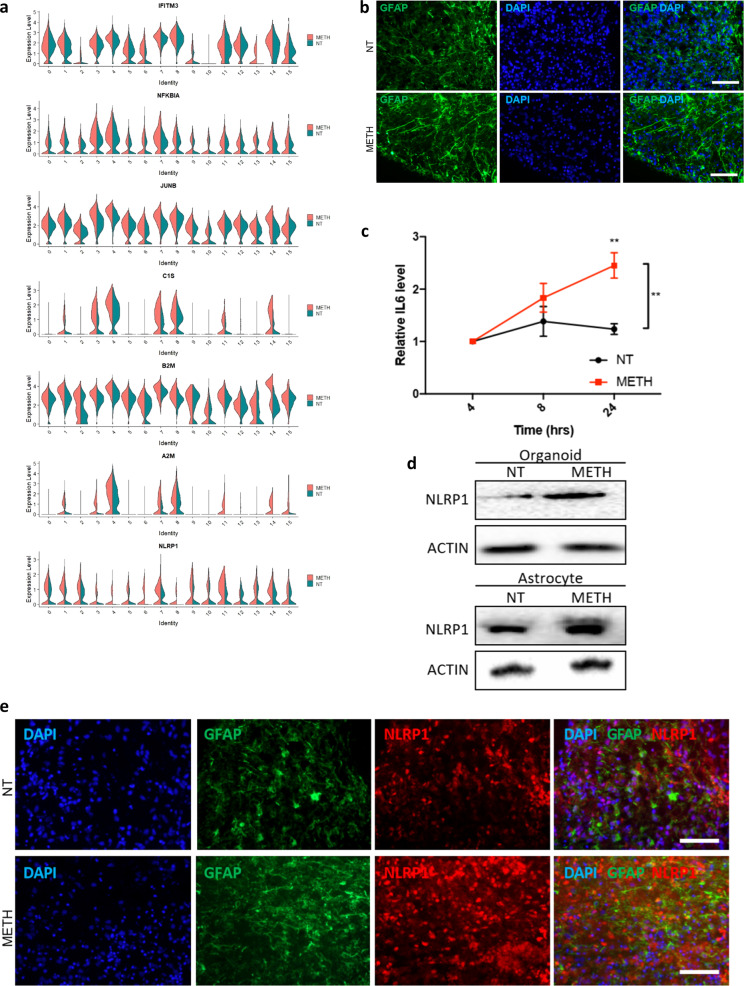
METH treatment induces neuroinflammation within cerebral organoids. **a** Violin plots showing scRNA-seq expression of immune response genes (*IFITM3*, *NFKBIA*), immediate early response genes (*JUNB*), inflammatory genes (*NLRP1*), complement factor (C1S), and factors regulating immune system (*B2M* and *A2M*) genes in all clusters in control (NT) and METH-treated (METH) organoids. **b** Untreated (top row) and METH-treated (bottom row) cerebral organoids immunostained for astrocyte marker GFAP (green) and DAPI (blue). Scale bar represents and 100 µm. **c** Relative expression of IL-6 as measured by ELISA. Supernatant from control and METH-treated organoids was collected at 4, 8, and 24 h post treatment and analyzed by ELISA. IL-6 expression levels were normalized to early 4 h expression levels to account for differences in organoid volume and heterogeneity. **p value < 0.01. **d** Immunoblot of control (NT) and METH-treated whole organoid lysates (top) and monolayer neural stem cell-derived astrocytes (bottom) for NLRP1 and GAPDH after 1 week of treatment. **e** Untreated (top row) and METH-treated (bottom row) cerebral organoids immunostained for astrocyte marker GFAP (green), inflammasome component NLRP1 (red), and DAPI (blue). Scale bar represents 100 µm.

Because glial cells, such as oligodendrocytes and astrocytes, regulate neuronal and synaptic functions, neural–glial and neuroimmune interactions represent a potentially important mechanism through which drugs may alter synaptic plasticity [28, 29]. The upregulation of immediate early response genes (*JUN, FOS, IER2*, and *IER3*), complement factors (*C1S, C1R*), cytokines (*CXCL8*), and factors playing role in immune system such as B2M and A2M suggested that organoids may be immunocompetent, and that METH induces neuroinflammation within organoid astrocytes (Fig. 3a–d and S3). To determine whether the upregulation of immediate early genes, immune response related genes, and complement factors were specifically upregulated in astrocytes or upregulated across all cell types in response to METH treatment, we analyzed *IFITM3*, *NFKBIA, JUNB, C1S, B2M, A2M*, and *NLRP1* expression across all clusters in METH-treated and control cells (Fig. 4a and S4A, B). These analyses revealed that, in general, METH upregulated immediately early genes, apoptotic genes, immune response genes, and complement factors across most cell types (Fig. 3a–d and S3). These data suggest that cerebral organoids may be utilized to model diseases which have a neuroimmune or neuroinflammatory etiology or pathology.

To validate these scRNA-seq findings, the neuroinflammatory response of METH-treated organoids was assessed at the protein level. During neuroinflammation, astrocytes are activated, resulting in morphological and phenotypic changes [30]. Thus, we first evaluated the extent of astrogliosis within METH-treated organoids by immunostaining for GFAP (Fig. 4b). During METH treatment, organoids exhibited both an increase in GFAP expression and thicker GFAP+ filaments (Fig. 4b), suggesting that METH treatment activated cerebral organoid derived astrocytes.

During astrogliosis, activated astrocytes express elevated levels of cytokines and chemokines [30]. Thus, we then analyzed the time-dependent expression of IL-6, a known pro-inflammatory cytokine triggered by drug abuse [29], by enzyme-linked immunosorbent assay (ELISA) (Fig. 4c). As suspected, cerebral organoids treated with METH expressed elevated levels of IL-6 relative to its steady state expression in a time-dependent manner.

**Fig. 5:**
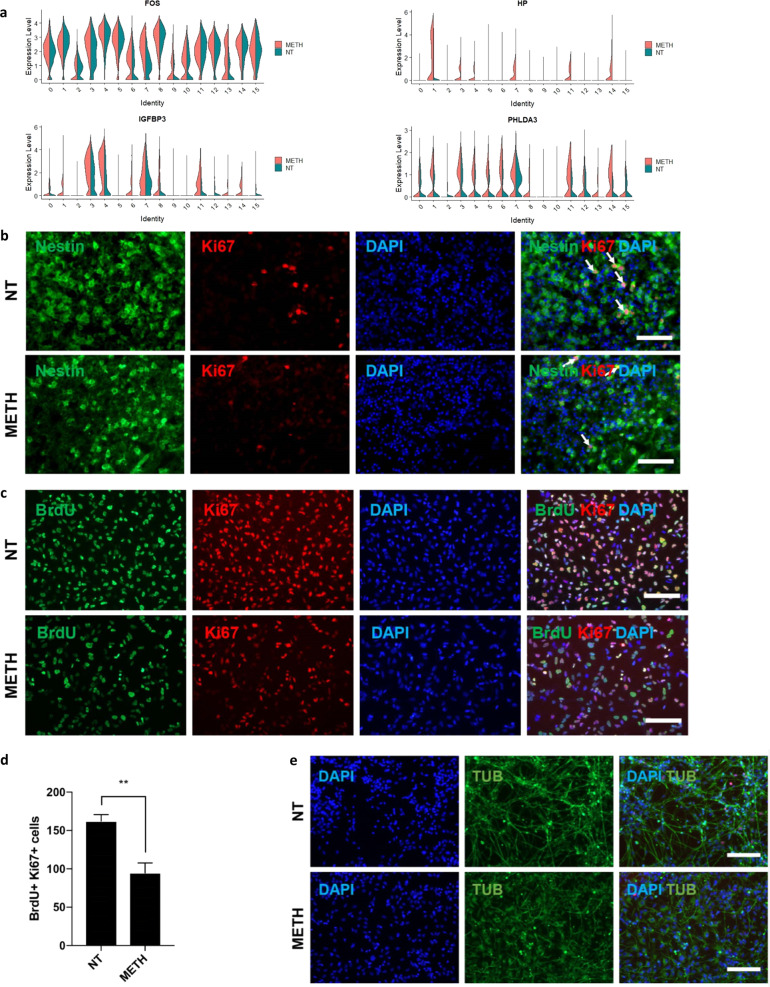
METH treatment attenuates neural stem cell proliferation and differentiation. **a** Violin plots showing scRNA-seq expression of *FOS, IGFBP3, HP*, and *PHLDA3* in all clusters in control (NT) and METH-treated (ME) organoids following 1 week of treatment. **b** Untreated (top row) and METH-treated (bottom row) cerebral organoids immunostained for neural stem cell marker Nestin (green), proliferation marker Ki67 (red) and DAPI (blue) after 1 week of treatment. Scale bar represents and 100 µm. **c** Untreated (top row) and METH-treated (bottom row) monolayer neural stem cells immunostained for BrdU (green), proliferation marker Ki67 (red) and DAPI (blue) after 1 week of treatment with 5 µM METH. Scale bar represents and 100 µm. **d** Quantification of BrdU+ Ki67+ neural stem cells in five fields at 20×. **e** Untreated (top row) and METH-treated (bottom row) monolayer neural stem cells immunostained for neuronal marker b-tubulin (green) and DAPI (blue) after 2 weeks of treatment with 5 µM METH during neural induction and neuronal differentiation. Scale bar represents 100 µm.

In addition, scRNA-seq data revealed an upregulation of NLRP1, a component of the inflammasome complex that may instigate the release of pro-inflammatory cytokines and activation of caspases (Fig. 4e). Immunostaining for NRLP1 expression in organoids revealed both an increase in GFAP expression as well as increased NRLP1, suggesting a neuroinflammatory reaction to METH treatment (Fig. 4e). In addition, METH treatment increased NLRP1 expression in lysates from whole organoids treated with METH and lysates from monolayer NSC-derived astrocyte cultures (Fig. 4d). In all, these data suggest that METH treatment induced a neuroinflammatory response in astroglial cells within cerebral organoids, thus providing evidence that organoid derived-glial cells are functionally active. METH induces cell cycle arrest and dysregulates differentiation. In addition to increased immediate early gene, apoptotic genes, complement factor, and neuroinflammatory gene expression following METH treatment, we observed an increase in insulin like growth factor binding protein-3 (IGFBP-3), FOS, and cell cycle regulator PHLDA3 expression in various neuronal cell types including astrocytes (Fig. 5a). Previous studies have shown that METH treatment enhanced embryonic oxidative DNA damage, thereby resulting in neurodevelopment deficits [31]. In addition, prior studies highlighted the role of IGFBP-3, PHLDA3, and FOS in the regulation of neural progenitor cell proliferation, differentiation, cell cycle, and cell survival pathway Akt [32–36]. Thus, we analyzed the effect of METH on IGFBP-3 expression and potential cell cycle arrest during NSC proliferation and differentiation. METH increased the expression of IGFBP-3, FOS, and PHLDA3 across almost all cell types within cerebral organoids (Fig. 5a). Thus, we hypothesized that the effect of insulin like growth factor-1 plays an important role in the proliferation and differentiation of neural progenitor cells and would be most impactful on symmetrically and asymmetrically dividing NSCs during embryonic development. NSCs in cerebral organoids treated with METH exhibited decreased proliferation as evidenced by a decrease in Ki67+ Nestin+ cells (Fig. 5b). These findings were validated in BrdU treated, homogeneous, monolayer NSC cultures treated with METH for a week (Fig. 5c, d and S5). Both BrdU+ and Ki67+ NSCs were significantly decreased.

To determine the impact of METH-induced cell cycle arrest on brain development, we analyzed the effect of METH in NSCs during neuronal differentiation. METH-treated NSCs exhibited a decrease in b-tubulin + neuron following 2 weeks post neuronal induction (Fig. 5e). These findings highlight the negative impact of METH treatment during neurodevelopment and neuronal differentiation.

## Discussion

“Intrinsic” cerebral organoids are a unique model system in which human cell type specification, self-organization, and heterogeneous intercellular communication occur simultaneously in an undirected manner in vitro. While previous publications have utilized scRNA-seq to classify cellular diversity within cerebral organoid models [15, 17] and others have utilized pseudotemporal ordering to analyze the transcriptional programs which control neuronal differentiation [19, 37–39], the majority of these studies have focused on the NSC and neuronal populations within 3D cerebral organoid models. Yet, astrocytes are also functionally diverse and are the largest class of glial cells in the mammalian brain. Functionally, they are implicated in trophic support, maintaining blood brain barrier, regulating neurogenesis, as well as metabolic support to neurons [20, 40]. Moreover, there are many types of astrocytes classified based on the morphology and expression of specific proteins in rodents, human, and nonhuman primates. Various signaling molecules such as MAPKs, NF-kB, and JAK-STAT regulate the gene expression profile in astrocytes in numerous physiological states and contribute to the generation and maintenance of glial diversity. Thus, here we utilized scRNA-seq to characterize cerebral organoids and study the effects of prenatal METH exposure on fetal brain development using cerebral organoid models. Moreover, this is, to the best of our knowledge, the first use of single-cell RNA-seq to evaluate glial cell diversity and the first example of neuro-immunity and neuroinflammation within cerebral organoids.

Here we utilized METH as a model system in which neuronal, NSC, and glial cells are adversely affected in cell-type-specific manners. Organoids were treated with METH over a 1-week-period to study the effect of prenatal METH exposure on the developing brain and scRNA-seq was utilized to identify differentially expressed genes following METH treatment.

We observed that astroglial cell types exhibited a robust transcriptional response when exposed to METH treatment. Astrocytes provide trophic support for neurons and surrounding cells and are involved in the regulation of transmission and synaptic activity, thus they represent a crucial component of the central nervous system when studying METH-related neurotoxicity and particularly cytokine and immune dysregulation, yet they are often overlooked [1, 27, 41].

Because astrocytes and other glial cells are integral in regulating synaptic plasticity and modulating the neuroimmune response during drug treatment, we analyze cytokine and neuroinflammatory marker expression at the RNA and protein level by scRNA-seq, ELISA, immunohistochemistry, and immunoblotting. In all, these data confirm that METH treatment elicits gliosis and neuroinflammation within cerebral organoids as indicated by enhanced IL-6 expression, overexpression of GFAP and NLRP-1. Consistent with our findings, it was recently found that neuroinflammation was induced in primary mice astrocytes and astrocytic cell lines treated with METH through the involvement of Toll-Like Receptor 4, caspase 11, and nuclear factor-Kappa B [42–44]. The induction of neuroinflammation resulted in neurotoxicity by upregulating cytokines IL-6 and IL-8 levels [42, 44]. Thus, this is, to the best of our knowledge, the first example showing cerebral organoids modeling neuroinflammation, thus making cerebral organoids a relevant model system for a range of diseases in which neuroinflammation contributes to pathogenesis.

In addition, cAMP signaling and glutamate regulation in the astrocyte population is disrupted by differential expression of *FOS*, *DUSP1*, and *JUN* which has potential neurotoxic implications [45]. JUN and FOS form the AP-1 early response transcription factor well known to regulate the cellular response to drugs in addition to cell growth, differentiation, and apoptosis [46, 47]. As such, JUN and FOS play a key role in mediating METH-induced neurotoxicity [46]. Our analyses have placed well-established regulators of METH addiction, including *FOS* and *JUN*, *PHLDA3* and *IGFBP-3* into cell-type-specific expression networks with previously unestablished genes, suggesting a potential co-regulatory or causal relationship for future validation and therapeutic targeting.

In all, here we showed that organoid models, which are composed of a diverse range of glial, neuronal, and NSC types, are a powerful model system for analyzing the effects of drugs and neuroinflammatory disorders in the brain when coupled with scRNA-seq. Through these assays, we identified novel drug-induced gene correlation networks and established cerebral organoids as a physiologically relevant model system for studying complex neuroinflammatory diseases.

## Materials and methods

### Materials

#### Cells

H9 hESCs feeder-dependent and feeder-independent (from Wicell Research Institute, cat. No. WA09), H9 hESCs derived Human NSCs (hNSCs) were purchased from Gibco (cat. No. N7800200).

#### Growth media and supplements

DMEM-F12 medium (cat. no. 11320082), StemPro® NSC SFM—Serum-Free Human Neural Stem Cell Culture Medium (cat. No. A1050901), knockout serum replacement (KOSR, cat. no. 10828–028), MEM nonessential amino acids (MEM-NEAA, cat. No. 11140050). N_2_ supplement (cat no.17502048), B-27 supplement without vitamin A (cat. No. 12587010), B-27 supplement with vitamin A (cat. No. 17504044), glutamine/glutaMAX (200 mM, cat. No. 35050061, A2916801), Dispase (cat no. 17105–0412q), and Collagenase IV (cat. No. 17104019) were obtained from Invitrogen (USA). Fetal bovine serum (FBS, cat. No. 10438–018) and bovine serum albumin (BSA, BP9703–100) obtained from Fischer Scientific (Gibco, Invitrogen, USA). Heparin (cat. No. H3149), basic fibroblast growth factor (bFGF, F0291), and epidermal growth factor (EGF, E9644) were procured from Sigma-Aldrich (USA) while mTeSR1 medium (Cat. No. 05850) was procured from Stem cell Technologies (USA).

#### Chemicals and antibodies

METH (cat. No. M8750), 5′-bromo-2′-deoxyuridine (BrdU) (cat. No. B5002), beta-mercaptoethanol (cat. No. M7522), and primary antibody GFAP (anti-mouse, Sigma, G3893) were purchased from Sigma Aldrich (USA). Protein IP lysis buffer (cat. No. 87787) and protease and phosphatase inhibitors cocktail (Cat. No. A32955) was obtained from Pierce (USA) while Bio-Rad DC protein assay kit (Cat. No. 5000112) and chemiluminescence substrate (cat. No. 34580) were purchased from Thermo Scientific (USA). Optimal cutting temperature (OCT) compound (cat. No. 4583) was procured from Tissue-Tech (USA). Primary antibodies such as Ctip2 (anti-rat, cat. No. ab18465), Sox2 (anti-rabbit, ab97959), β-Tubulin III (anti-mouse, ab7751–100), and Nestin (anti-chicken, ab134017) were obtained from Abcam, (USA). Ki67 (anti-rabbit, Millipore AB9260), and PVDF membrane were obtained from Chemicon (Millipore, Billerica, MA, USA). Rabbit anti-β-actin (cat. No. 4970) and Nlrp1 (anti-rabbit, cat. No. 4990) primary antibodies were obtained from Cell Signaling Technology (Danvers, MA, USA), while mouse anti-BrdU (cat. No. sc-32323) primary antibodies was procured from Santa Cruz Biotechnology (Santa Cruz, CA, USA). Alexa Fluor 488, 594, and 647 conjugated secondary antibodies were purchased from Molecular Probes (Invitrogen, USA). Anti-fade mounting medium with DAPI was obtained from Vector Labs (Vectashield, Vector Laboratories, CA).

### Cell culture and organoid generation

All studies were conducted in accordance with approved IRB protocols by the University of California, San Diego. H9 hESCs (WA09) from WiCell was cultured on a feeder layer of irradiated mouse embryonic fibroblasts or under feeder-free matrigel coated conditions following previously established protocols. Feeder-dependent H9 hESCs were detached from their feeder layer using 1 mg/ml collagenase (dissolved in DMEM-F12 medium) for 15–20 min at 37 °C in CO_2_ incubator and 0.5 mg/ml dispase (dissolved in DMEM-F12 medium) for an additional 15 min at 37 °C in CO_2_ incubator. Wells were washed with media to collect floating undifferentiated hESCs and colonies were dissociated using Accumax or Versene solution at 37 °C for 10 min to generate a single-cell suspension. At day 0, embryoid bodies were formed using the hanging drop method with 4500 cells/drop in DMEM/F12 media supplemented with 20% knockout serum replacement (KOSR), bFGF (4 ng/ml), MEM nonessential amino acids (MEM-NEAA, 1%, vol/vol), and glutamine (200 mM, 1%, vol/vol) or grown in microwell plates. After 2 days of hanging drop culture, embryoid bodies were transferred to sterile petri dishes with refreshed media. After 6 days in culture, embryoid bodies were transferred to new petri dishes containing neural induction media consisting of DMEM/F12, N2 supplement (1%, vol/vol), MEM-NEAA (1%, vol/vol), glutamine (200 mM, 1%, vol/vol), and heparin (1 μg/ml) until day 11. At day 11, organoids were transferred to Matrigel droplets (30 μl) and cultured in 1:1 mixture of DMEM/F12 and Neurobasal medium supplemented with B-27 without vitamin A (1%, vol/vol), N2 (1%, vol/vol), NEAA (1%, vol/vol), insulin, beta-mercaptoethanol, and glutamine (200 mM, 1%, vol/vol). Twenty organoids were then transferred to stir flask bioreactors (125 ml) containing magnetic shaft and stirring speed was maintained 50–60 rpm. For long term growth on day 15 in 75–100 ml of cerebral organoid differentiation media with vitamin A. Media was changed every 3 days. Organoids were treated with 5 μM METH dissolved in organoid culture media every other day for 1 week before fixing for immunostaining or dissociation for scRNA-seq.

### NSC culture

Human NSCs derived from H9 hESCs were purchased from Gibco (N7800200) and cultured in Knockout D-MEM/F-12 media containing 2 mM GlutaMax (1%, vol/vol), 20 ng/ml bFGF, 20 ng/ml EGF, and 2% StemPro Neural Supplement (vol/vol) on Matrigel- or CELLStart-coated plates.

### Astrocyte differentiation

NSCs were plated on Matrigel coated plates in complete Stem-Pro NSC SFM medium at 2.5 × 10^4^ cells/cm^2^. After 2 days of NSC culture, the media will be changed to astrocyte differentiation medium which consists of D-MEM supplemented with N-2 (1%, vol/vol), GlutaMAX-I (2 mM, 1%, vol/vol), FBS (1%, vol/vol), and Antibiotic-Antimycotic solution (1%, vol/vol) every 3–4 days for 1 month.

### Neuronal differentiation

To differentiate NSCs into the neurons, hNSCs were plated in polyornithine and laminin-coated culture dishes in complete StemPro NSC SFM at 2.5–5 × 10^4^ cells/cm^2^. After 2 days, the media was changed to neural differentiation medium consisting of neurobasal medium supplemented with B-27 serum-free supplement (1×, vol/vol), GlutaMAX-I (200 mM, 1×, vol/vol), and antibiotic -antimycotic (1×, vol/vol) solutions and every 3–4 days for 2 weeks.

### Immunohistochemistry

To section and stain organoids, organoids were washed with phosphate buffer saline (PBS) and incubated in cell recovery solution for 30 min to dissolve the surrounding Matrigel. Cell recovery solution is used to recover cells or organoids from matrigel matrix for subsequent sectioning and biochemical analyses. Organoids were washed with PBS and then fixed with 4% paraformaldehyde (PFA) for 1 h. Fixed organoids were washed three times with PBS, stained with hematoxylin for 5 min and incubated in 30% sucrose overnight. Sucrose solution was removed, organoids were washed with PBS and embedded in OCT compound for cryosectioning. We typically cut 20-μm-thick sections of organoid for immunohistochemical analysis using cryostat. Cryosections were blocked in 5% BSA in PBS for 1 h, washed three times with PBS + 0.1% Tween-20 (PBST), and incubated at 4 °C overnight with primary antibodies: Ctip2 (1:500, Rat, Abcam ab18465), Sox2 (1 μg/ml, Rabbit, Abcam ab97959), GFAP (1:500, Mouse, Sigma, G3893), β-Tubulin III (1:500, mouse, Abcam ab7751–100), Ki67 (1:200, Rabbit, Millipore AB9260), Nestin (1:1000, Chicken, Abcam ab134017), and Nlrp1(1:200, Rabbit, Cell Signaling Technology (CST)-4990). Cryosections were washed three times with PBS with 0.1% Tween-20 (PBST) to remove primary antibody 1 h prior to secondary antibody incubation. We used alexa fluor conjugated secondary antibodies including anti-Mouse alexa fluor 488, 594, anti-chicken alexa fluor 488, 647, and anti-rabbit alexa fluor 488, 594 (Molecular Probes, USA, 1 μg/ml dilution in PBS). Cryosections were washed three more times to remove secondary antibody before being mounted with Vectashield hardset mounting medium with nuclear stain (DAPI) following manufacturer’s instructions.

### BrdU immunocytochemistry

BrdU is a thymidine analog that is incorporated into DNA during the S phase of the cell cycle and labels proliferating cells. To assess cell proliferation, control, and METH-treated human H9 derived NSC cultures and organoids were treated with BrdU (Sigma Aldrich B5002, 20 µM) for 12 h before fixing cells with 4% PFA and processing for BrdU immunocytochemistry. In brief, denature DNA of organoids sections/hNSCs with 2 N HCl for 10 min at 37 °C followed by neutralization with borate buffer (0.1 M, pH 8.5) for 10 min at room temperature. After blocking the cells for 30 min at room temperature, we used primary mouse antibodies for BrdU at 1:200 dilution in blocking buffer (BrdU, sc-32323, anti-mouse, 1:200) for overnight. The following day, wash and incubate cells with Alexa fluor 488 anti-mouse secondary labeling (1 μg/ml diluted in PBS) and counterstain with nuclear stain Hoechst 33342.

### Immunoblotting and ELISA

Cerebral organoids (~50 mg) or astrocytes were lysed in 500 μl of Pierce IP lysis buffer containing protease inhibitor cocktail (Roche) and proteins were extracted by centrifugation at 13,000 × *g* for 10 min at 4 °C. The concentration of extracted proteins was determined by Bio-Rad DC protein assay kit as per manufacturer instructions (Bio-Rad) and equal amounts of protein (25 μg) were resolved by SDS-PAGE and transferred to PVDF membranes (Bio-Rad). Membranes were blocked with 5% nonfat milk in PBST or Thermo Scientific Pierce Fast Western Blot Kits followed by overnight incubation at 4 °C with primary antibodies such as β-Actin (1:1000, Cell Signaling Technology (CST)-4970) and NLRP1 (1:1000, CST-4990). On following day, membranes were then washed three times with tris-buffered Saline with 0.1% Tween-20 (TBST) and incubated for 2 h with secondary antibody conjugated with horseradish peroxidase. Immunoreactive protein signals were detected with Super-signal West Pico Chemiluminescent Substrate (Pierce, USA). Densitometric analysis of protein bands were carried out by using Image Lab software (Bio-Rad, USA) and the values of proteins were normalized with β-Actin levels.

ELISA was performed using the Human IL-6 ELISA kit (eBioscience) following manufacturer’s instructions. Briefly, ELISA plates were incubated with IL-6 capture antibody overnight in coating buffer. Wells were washed with 0.05% PBST five times and blocked with Assay Diluent for 1 h at room temperature. After five additional washes, organoid supernatant collected at 4, 8, and 24 h post-METH treatment and IL-6 standards were added to wells and incubated overnight at 4 °C. The following day, wells were washed five times with PBST, incubated with diluted detection antibody for 1 h at room temperature. Wells were again washed five times before diluted Avidin-HRP was added for 30 min at room temperature. After seven washes, substrate solution was added for 15 min, followed by stop solution. ELISA plates were read by a Synergy 2 plate reader (Biotek) at 450 nm and 570 nm.

### 10x Chromium scRNA-seq

scRNA-seq was performed on eight untreated and six METH-treated organoids. Organoids were manually dissociated into single-cell suspension using the Milteny Biotec Neural Tissue Dissociation Kit (P) (Milteny Biotec, 130–092–628) following manufacturer’s instructions. Briefly, organoids were washed with PBS and cut into small pieces using a scalpel. Tissues were resuspended in warmed enzyme mix 1 containing papain and rotated for 15 min at 37 °C. Enzyme mix 2 was added and tissues were mechanically dissociated using a wide-tipped pipet. Cell suspensions were passed through a 40 μm cell strainer and centrifuged to pellet. Cell concentrations and viability were assessed by trypan blue staining.

scRNA-seq libraries were generated using the 10x Genomics Chromium Single-cell 3′ Library & Gel Bead Kit V2 (10x Genomics, PN-120237) at the UCSD Institute for Genomic Medicine Genomic Center. Libraries were generated from organoid single-cell suspensions following manufacturer’s protocol. Briefly, single-cell suspensions were mixed with RT primer and enzyme master mixes and loaded into the Single-cell 3′ Chip (10x Genomics, PN-120236) along with single-cell 3′ gel beads, and partitioning oil to generate GEMs using the Chromium Controller. GEMs were transferred and reverse transcribed to generate cDNA. After RT, cDNA was recovered, cleaned, and amplified. To construct the library, cDNAs were processed by fragmentation, end repair, and A-tailing. Libraries were size selected using SPRI-Select beads. P5 and P7 adapters were ligated and libraries were again size selected using SPRI-Select beads. Final libraries were analyzed using the Agilent TapeStation system and quantified using Qubit (Invitrogen). scRNA-seq libraries were sequenced using a HiSeq4000.

### Bioinformatic analysis

Chromium single-cell 3′ RNA-seq libraries were aligned to GRCh38.p11 using the 10x Genomics Cell Ranger 2.0.0 pipeline following manufacturer’s default settings. Briefly, alignment, filtering, and UMI counting was performed using cell ranger count (10x Genomics) and aggregated using cell ranger aggr. Tertiary analyses on cell ranger output files were performed using Seurat [48]. Data normalization, filtering, clustering, and differential gene expression analyses were performed using Seurat R package version 2.4 and 3.0 with R version 3.5.3 [25, 48]. Integrative single-cell analyses were performed following previously established Seurat pipelines with modifications [25]. Cells with fewer than 200 genes were filtered out. To integrate control and METH-treated samples, datasets were aligned using highly variable genes within samples with the Run Multi CCA and Align Subspace functions (Seurat V2.4). TSNE clustering was used to determine the clusters of the cells. Differential gene expression analyses were performed by subsetting the data based on cluster identity and assuming a negative-binomial distribution. Treatment response to determine up and down differentially expressed genes due to METH treatment was determined by finding the markers between all of the cells between the two treatment conditions outlined in the immune alignment tutorial for Seurat. The list of variable genes was separated into two separate gene lists based on up and down regulation. The entire list of expressed genes was used to create a reference gene list for GO analysis via PANTHER for overrepresentation analysis (http://geneontology.org/). The up and downregulated genes were uploaded into PANTHER separately and the results from the two analyses were sorted based on raw *p* value. The log of the raw *p* value of these results was calculated and the top 9 downregulated pathways and top 20 upregulated pathways were used with the ggplot2 package version 3.1.1 in R to create Fig. S2. All dot plots and violin plots were created using functions from the Seurat package version 3.0.

## Supplementary information

Supplementary figures and legends

## References

[1] Prakash MD, Tangalakis K, Antonipillai J, Stojanovska L, Nurgali K, Apostolopoulos V (2017). Methamphetamine: effects on the brain, gut and immune system. Pharmacol Res.

[2] Hsieh JH, Stein DJ, Howells FM (2014). The neurobiology of methamphetamine induced psychosis. Front Hum Neurosci.

[3] Gonzales R, Mooney L, Rawson RA (2010). The methamphetamine problem in the United States. Annu Rev Public Health.

[4] Chang L, Alicata D, Ernst T, Volkow N (2007). Structural and metabolic brain changes in the striatum associated with methamphetamine abuse. Addiction.

[5] Du J, Quan M, Zhuang W, Zhong N, Jiang H, Kennedy DN (2015). Hippocampal volume reduction in female but not male recent abstinent methamphetamine users. Behav Brain Res.

[6] Smith LM, LaGasse LL, Derauf C, Grant P, Shah R, Arria A (2006). The infant development, environment, and lifestyle study: effects of prenatal methamphetamine exposure, polydrug exposure, and poverty on intrauterine growth. Pediatrics.

[7] Nguyen D, Smith LM, Lagasse LL, Derauf C, Grant P, Shah R (2010). Intrauterine growth of infants exposed to prenatal methamphetamine: results from the infant development, environment, and lifestyle study. J Pediatr.

[8] Chang L, Smith LM, LoPresti C, Yonekura ML, Kuo J, Walot I (2004). Smaller subcortical volumes and cognitive deficits in children with prenatal methamphetamine exposure. Psychiatry Res.

[9] Li B, Chen R, Chen L, Qiu P, Ai X, Huang E (2017). Effects of DDIT4 in methamphetamine-induced autophagy and apoptosis in dopaminergic neurons. Mol Neurobiol.

[10] Sun L, Li HM, Seufferheld MJ, Walters KR, Margam VM, Jannasch A (2011). Systems-scale analysis reveals pathways involved in cellular response to methamphetamine. PLoS ONE.

[11] Wongprayoon P, Govitrapong P (2017). Melatonin protects SH-SY5Y neuronal cells against methamphetamine-induced endoplasmic reticulum stress and apoptotic cell death. Neurotox Res.

[12] Lancaster MA, Renner M, Martin CA, Wenzel D, Bicknell LS, Hurles ME (2013). Cerebral organoids model human brain development and microcephaly. Nature.

[13] Eiraku M, Watanabe K, Matsuo-Takasaki M, Kawada M, Yonemura S, Matsumura M (2008). Self-organized formation of polarized cortical tissues from ESCs and its active manipulation by extrinsic signals. Cell Stem Cell.

[14] Mariani J, Coppola G, Zhang P, Abyzov A, Provini L, Tomasini L (2015). FOXG1-dependent dysregulation of GABA/glutamate neuron differentiation in autism spectrum disorders. Cell.

[15] Camp JG, Badsha F, Florio M, Kanton S, Gerber T, Wilsch-Brauninger M (2015). Human cerebral organoids recapitulate gene expression programs of fetal neocortex development. Proc Natl Acad Sci USA.

[16] Dang J, Tiwari SK, Lichinchi G, Qin Y, Patil VS, Eroshkin AM (2016). Zika virus depletes neural progenitors in human cerebral organoids through activation of the innate immune receptor TLR3. Cell Stem Cell.

[17] Quadrato G, Nguyen T, Macosko EZ, Sherwood JL, Min Yang S, Berger DR (2017). Cell diversity and network dynamics in photosensitive human brain organoids. Nature.

[18] Nowakowski TJ, Pollen AA, Di Lullo E, Sandoval-Espinosa C, Bershteyn M, Kriegstein AR. Expression analysis highlights AXL as a candidate Zika virus entry receptor in neural stem cells. Cell Stem Cell. 2016;18:591–6. 10.1016/j.stem.2016.03.012 PMC4860115 27038591

[19] Xiang Y, Tanaka Y, Patterson B, Kang YJ, Govindaiah G, Roselaar N (2017). Fusion of regionally specified hPSC-derived organoids models human brain development and interneuron migration. Cell Stem Cell.

[20] Ben Haim L, Rowitch DH (2017). Functional diversity of astrocytes in neural circuit regulation. Nat Rev Neurosci.

[21] Yu S, Zhu L, Shen Q, Bai X, Di X (2015). Recent advances in methamphetamine neurotoxicity mechanisms and its molecular pathophysiology. Behav Neurol.

[22] Auld DS, Robitaille R (2003). Glial cells and neurotransmission: an inclusive view of synaptic function. Neuron.

[23] Crews FT, Zou J, Qin L (2011). Induction of innate immune genes in brain create the neurobiology of addiction. Brain Behav Immun.

[24] Qian X, Nguyen HN, Song MM, Hadiono C, Ogden SC, Hammack C (2016). Brain-region-specific organoids using mini-bioreactors for modeling ZIKV exposure. Cell.

[25] Butler A, Satija R. Integrated analysis of single cell transcriptomic data across conditions, technologies, and species. 2017. 10.1101/164889. 10.1038/nbt.4096 PMC6700744 29608179

[26] Bortell N, Basova L, Semenova S, Fox HS, Ravasi T, Marcondes MC. Astrocyte-specific overexpressed gene signatures in response to methamphetamine exposure in vitro. J Neuroinflammation. 2017;14:49. 10.1186/s12974-017-0825-6 PMC5345234 28279172

[27] Jackson AR, Shah A, Kumar A (2014). Methamphetamine alters the normal progression by inducing cell cycle arrest in astrocytes. PLoS ONE.

[28] Cadet JL, Bisagno V. Glial-neuronal ensembles: partners in drug addiction-associated synaptic plasticity. Front Pharmacol. 2014;5:204. 10.3389/fphar.2014.00204 PMC4151032 25228881

[29] Lacagnina MJ, Rivera PD, Bilbo SD. Glial and neuroimmune mechanisms as critical modulators of drug use and abuse. Neuropsychopharmacology. 2017;42:156–77. 10.1038/npp.2016.121 PMC5143481 27402494

[30] Monnet-Tschudi F, Defaux A, Braissant O, Cagnon L, Zurich MG. Methods to assess neuroinflammation. Current protocols in toxicology. 2011; Chapter 12:Unit12 19. 10.1002/0471140856.tx1219s50 22058053

[31] Jeng W, Wong AW, Ting AKR, Wells PG. Methamphetamine-enhanced embryonic oxidative DNA damage and neurodevelopmental deficits. Free Radic Biol Med. 2005;39:317–26. 10.1016/j.freeradbiomed.2005.03.015 15993330

[32] Tseng CS, Chao HW, Huang HS, Huang YS (2017). Olfactory-experience- and developmental-stage-dependent control of CPEB4 regulates c-Fos mRNA translation for granule cell survival. Cell Rep.

[33] Kalluri HS, Dempsey RJ (2011). IGFBP-3 inhibits the proliferation of neural progenitor cells. Neurochem Res.

[34] Lu XF, Jiang XG, Lu YB, Bai JH, Mao ZB (2005). Characterization of a novel positive transcription regulatory element that differentially regulates the insulin-like growth factor binding protein-3 (IGFBP-3) gene in senescent cells. J Biol Chem.

[35] Apostolidis PA, Lindsey S, Miller WM, Papoutsakis ET (2012). Proposed megakaryocytic regulon of p53: the genes engaged to control cell cycle and apoptosis during megakaryocytic differentiation. Physiol Genom.

[36] Liao Y, Hung MC (2010). Physiological regulation of Akt activity and stability. Am J Transl Res.

[37] Rizvi AH, Camara PG, Kandror EK, Roberts TJ, Schieren I, Maniatis T (2017). Single-cell topological RNA-seq analysis reveals insights into cellular differentiation and development. Nat Biotechnol.

[38] Fletcher RB, Das D, Gadye L, Street KN, Baudhuin A, Wagner A (2017). Deconstructing olfactory stem cell trajectories at single-cell resolution. Cell Stem Cell.

[39] Dulken BW, Leeman DS, Boutet SC, Hebestreit K, Brunet A (2017). Single-cell transcriptomic analysis defines heterogeneity and transcriptional dynamics in the adult neural stem cell lineage. Cell Rep.

[40] Sofroniew MV, Vinters HV (2010). Astrocytes: biology and pathology. Acta Neuropathol.

[41] Cisneros IE, Ghorpade A (2014). Methamphetamine and HIV-1-induced neurotoxicity: role of trace amine associated receptor 1 cAMP signaling in astrocytes. Neuropharmacology.

[42] Du SH, Qiao DF, Chen CX, Chen S, Liu C, Lin Z (2017). Toll-like receptor 4 mediates methamphetamine-induced neuroinflammation through caspase-11 signaling pathway in astrocytes. Front Mol Neurosci.

[43] Du SH, Zhang W, Yue X, Luo XQ, Tan XH, Liu C (2018). Role of CXCR1 and interleukin-8 in methamphetamine-induced neuronal apoptosis. Front Cell Neurosci.

[44] Shah A, Silverstein PS, Singh DP, Kumar A (2012). Involvement of metabotropic glutamate receptor 5, AKT/PI3K signaling and NF-kappaB pathway in methamphetamine-mediated increase in IL-6 and IL-8 expression in astrocytes. J Neuroinflammation.

[45] Cisneros IE, Ghorpade A (2012). HIV-1, methamphetamine and astrocyte glutamate regulation: combined excitotoxic implications for neuro-AIDS. Curr HIV Res.

[46] Deng X, Jayanthi S, Ladenheim B, Krasnova IN, Cadet JL (2002). Mice with partial deficiency of c-Jun show attenuation of methamphetamine-induced neuronal apoptosis. Mol Pharm.

[47] Torres OV, McCoy MT, Ladenheim B, Jayanthi S, Brannock C, Tulloch I (2015). CAMKII-conditional deletion of histone deacetylase 2 potentiates acute methamphetamine-induced expression of immediate early genes in the mouse nucleus accumbens. Sci Rep.

[48] Satija R, Farrell JA, Gennert D, Schier AF, Regev A (2015). Spatial reconstruction of single-cell gene expression data. Nat Biotechnol.

