# Supplementary material for: Glial cell diversity and methamphetamine-induced neuroinflammation in human cerebral organoids
Source: Mol Psychiatry. 2020 Feb 12;26(4):1194–207. doi: 10.1038/s41380-020-0676-x (PMC7423603; doi:10.1038/s41380-020-0676-x)
Supplement: Supplementary file 1 — Supplementary figures and legends [file 41380_2020_676_MOESM1_ESM.pdf]

Supplementary Figure 1

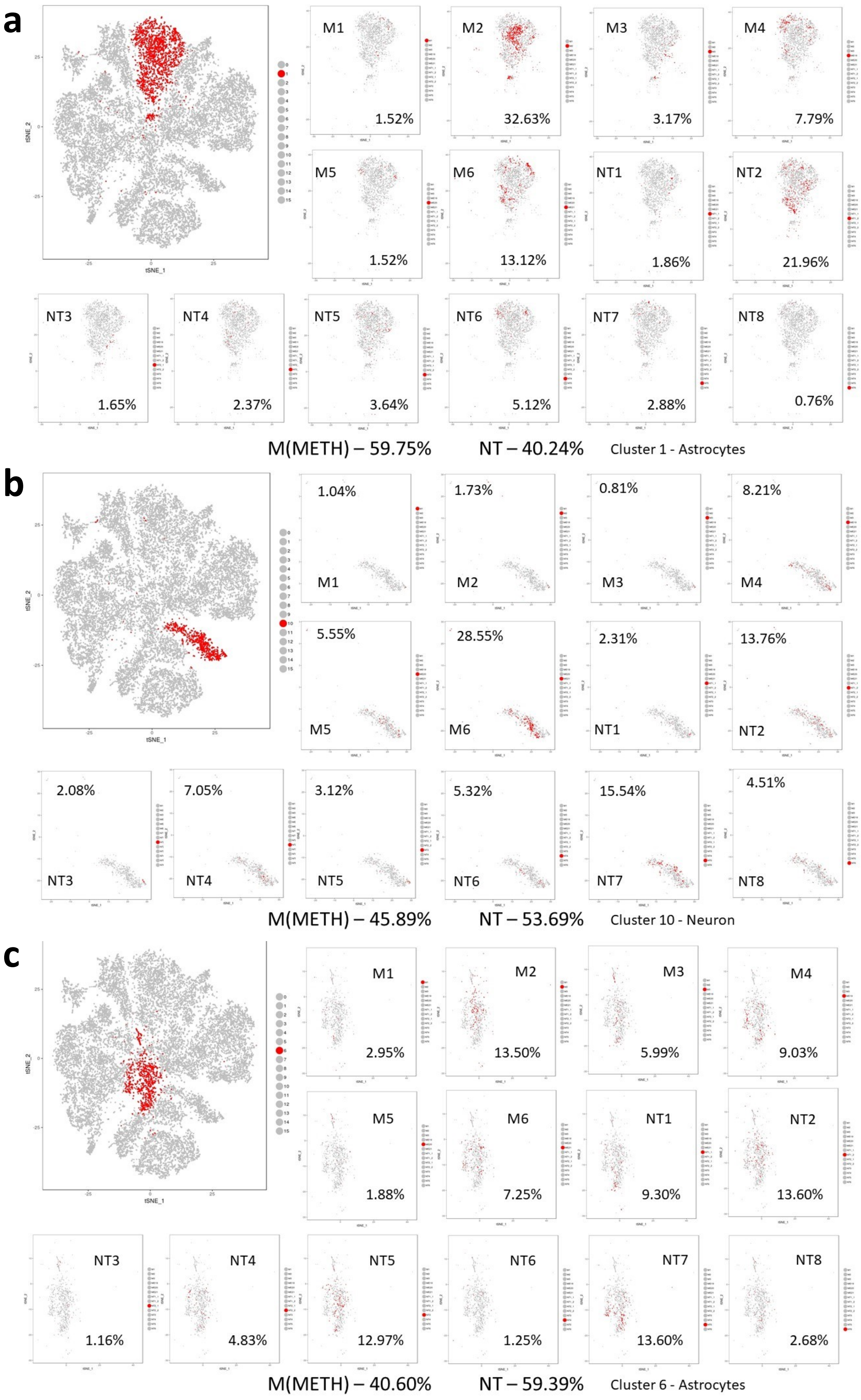

Supplementary Figure 1: Cerebral organoid heterogeneity. Related to Figure 1.

(a) Cluster 1, a cluster of astrocytes, with the composition of cells from each organoid from the two types of treatment and the overall percentage of cells from each treatment group to show the distribution of cells across treatment.

(b) Cluster 10, a cluster of neurons, with the composition of cells from each organoid from the two types of treatment and the overall percentage of cells from each treatment group to show the distribution of cells across treatment.

(c) Cluster 6, another cluster of astrocytes, with the composition of cells from each organoid from the two types of treatment and the overall percentage of cells from each treatment group to show the distribution of cells across treatment.

a

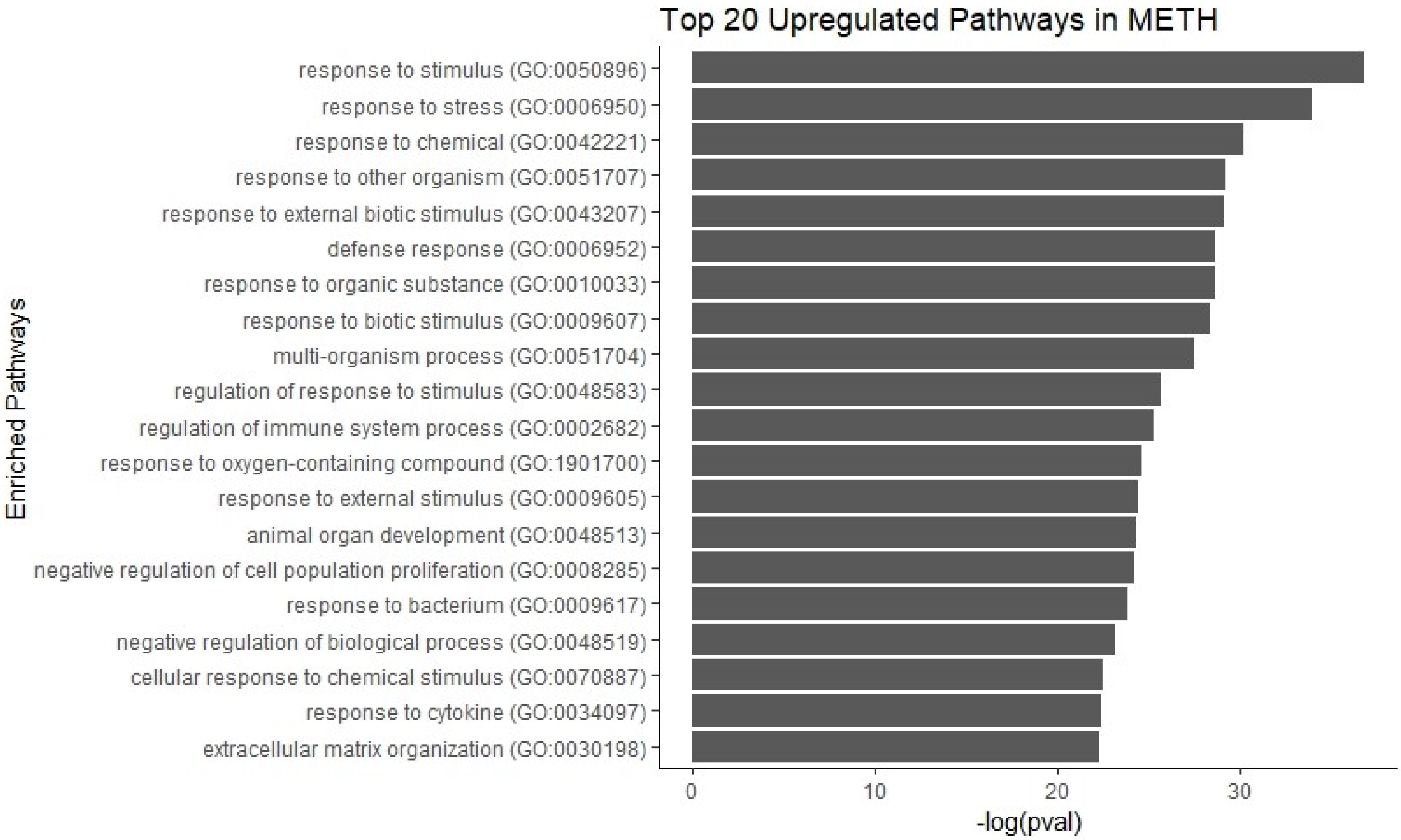
Top 20 Upregulated Pathways in METH
Enriched Pathways
response to stimulus (GO:0050896)
response to stress (GO:0006950)
response to chemical (GO:0042221)
response to other organism (GO:0051707)
response to external biotic stimulus (GO:0043207)
defense response (GO:0006952)
response to organic substance (GO:0010033)
response to biotic stimulus (GO:0009607)
multi-organism process (GO:0051704)
regulation of response to stimulus (GO:0048583)
regulation of immune system process (GO:0002682)
response to oxygen-containing compound (GO:1901700)
response to external stimulus (GO:0009605)
animal organ development (GO:0048513)
negative regulation of cell population proliferation (GO:0008285)
response to bacterium (GO:0009617)
negative regulation of biological process (GO:0048519)
cellular response to chemical stimulus (GO:0070887)
response to cytokine (GO:0034097)
extracellular matrix organization (GO:0030198)
0
10
20
30
-log(pval)

b

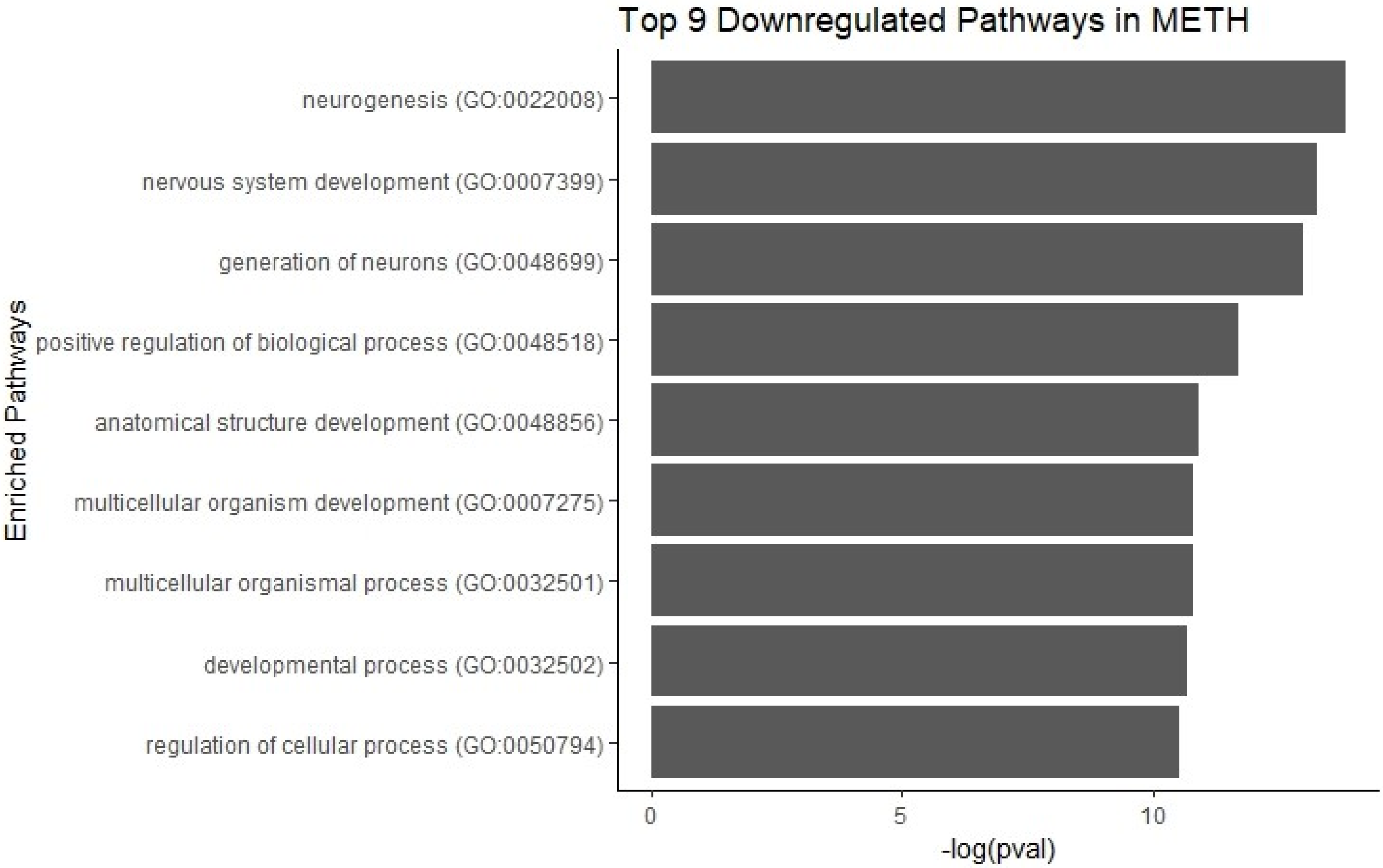
Top 9 Downregulated Pathways in METH
Enriched Pathways
neurogenesis (GO:0022008)
nervous system development (GO:0007399)
generation of neurons (GO:0048699)
positive regulation of biological process (GO:0048518)
anatomical structure development (GO:0048856)
multicellular organism development (GO:0007275)
multicellular organismal process (GO:0032501)
developmental process (GO:0032502)
regulation of cellular process (GO:0050794)
0
5
10
-log(pval)

Supplementary Figure 2: PANTHER pathways for up and down regulated genes due to METH treatment. Related to Figure 2.

(a) The top 20 upregulated pathways derived from the list of upregulated genes due to METH treatment via PANTHER. A total of 216 pathways were found to be upregulated.

(b) The top 9 downregulated pathways derived from the list of downregulated genes due to METH treatment via PANTHER.

a

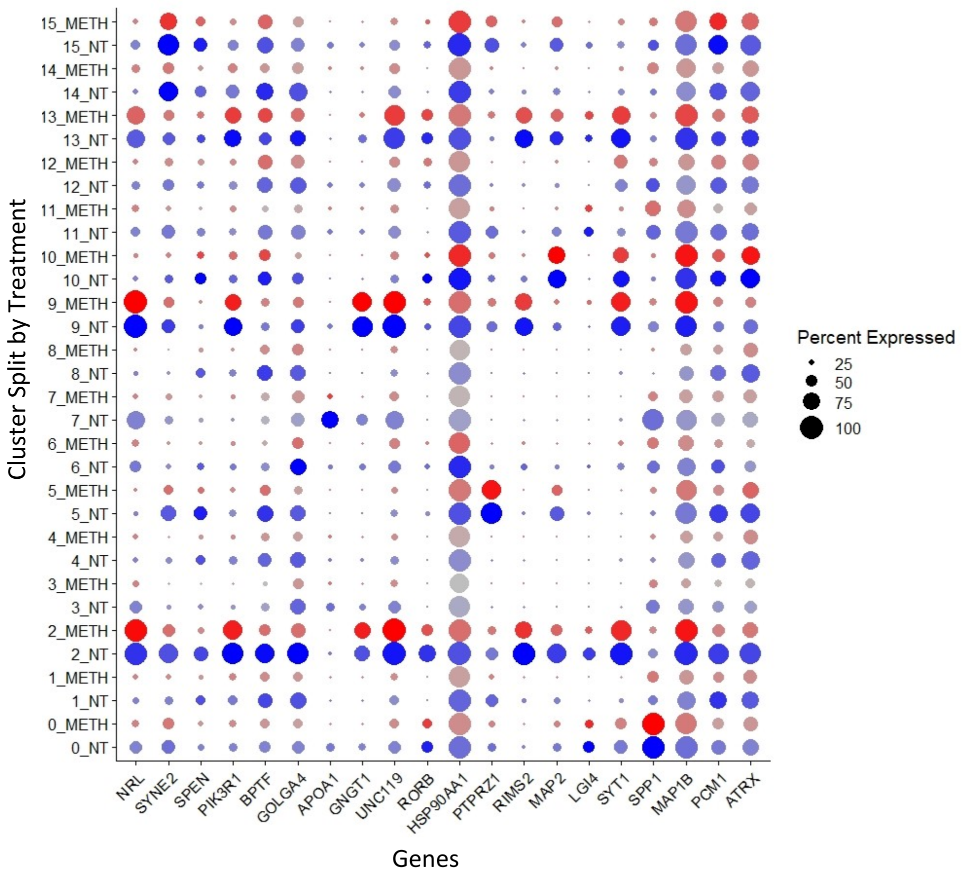
15_METH
15_NT
14_METH
14_NT
13_METH
13_NT
12_METH
12_NT
11_METH
11_NT
10_METH
10_NT
9_METH
9_NT
8_METH
8_NT
7_METH
7_NT
6_METH
6_NT
5_METH
5_NT
4_METH
4_NT
3_METH
3_NT
2_METH
2_NT
1_METH
1_NT
0_METH
0_NT
Cluster Split by Treatment
NRL
SYNE2
SPEN
PIK3R1
BPTF
GOLGA4
APOA1
GNGT1
UNC119
RORB
HSP90AA1
PTPRZ1
RIMS2
MAP2
LGI4
SYT1
SPP1
MAP1B
PCM1
ATRX
Genes
Percent Expressed
25
50
75
100

b

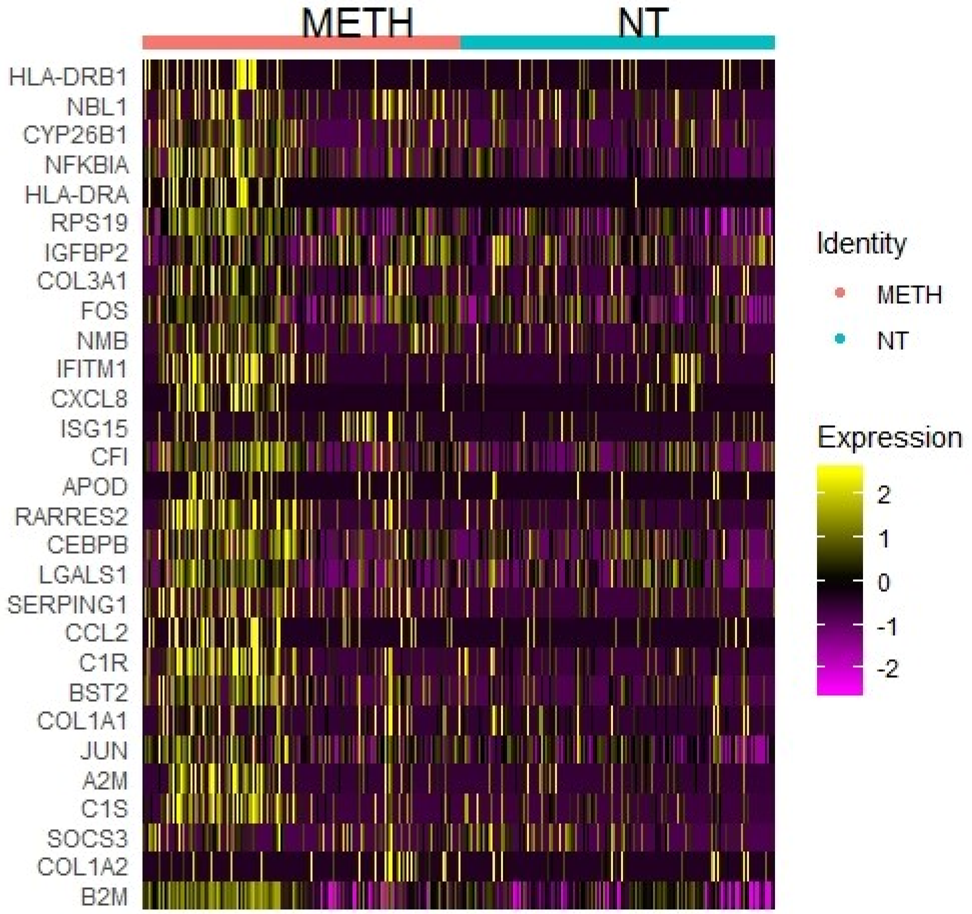
METH
NT
HLA-DRB1
NBL1
CYP26B1
NFKBIA
HLA-DRA
RPS19
IGFBP2
COL3A1
FOS
NMB
IFITM1
CXCL8
ISG15
CFI
APOD
RARRES2
CEBPB
LGALS1
SERPING1
CCL2
C1R
BST2
COL1A1
JUN
A2M
C1S
SOCS3
COL1A2
B2M
Identity
METH
NT
Expression
2
1
0
-1
-2

Supplementary Figure 3: Downregulation of genes pertaining to neurogenesis as a result of METH treatment. Related to Figure 3.

(a) A dot plot of the genes from the set of genes downregulated by METH (Figure 2A) treatment identified by PANTHER to be related to neurogenesis, generation of neurons, and nervous system development. Each cluster is divided by treatment, where blue dots represent cells from control organoid and red dots represent cells from METH treated organoids. Darker shades of blue and red and size of dots represent greater expression of genes and percentage of cells expressing the gene.

(b) A heatmap of the same genes from Figure S3A separated by treatment, for a holistic view of the data.

Supplementary Figure 4

a

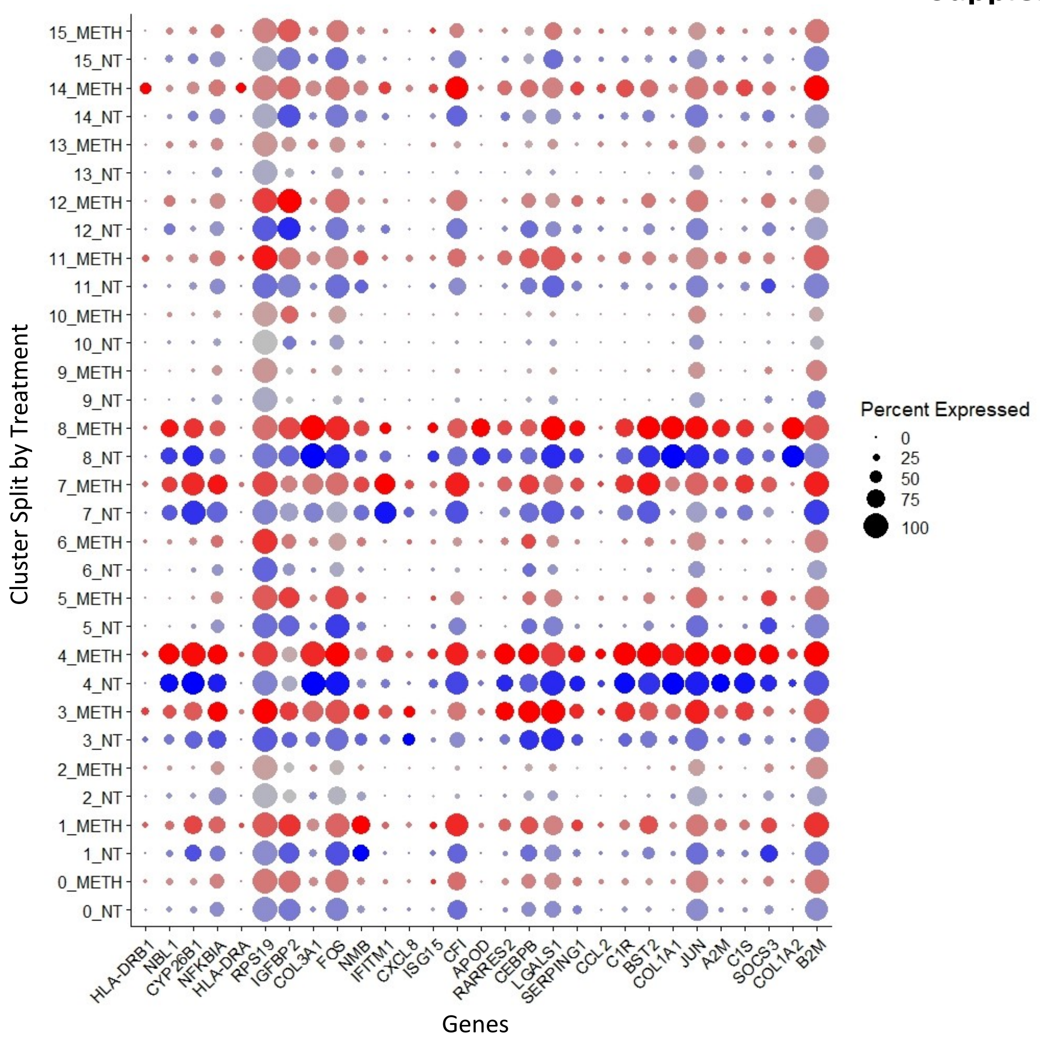

b

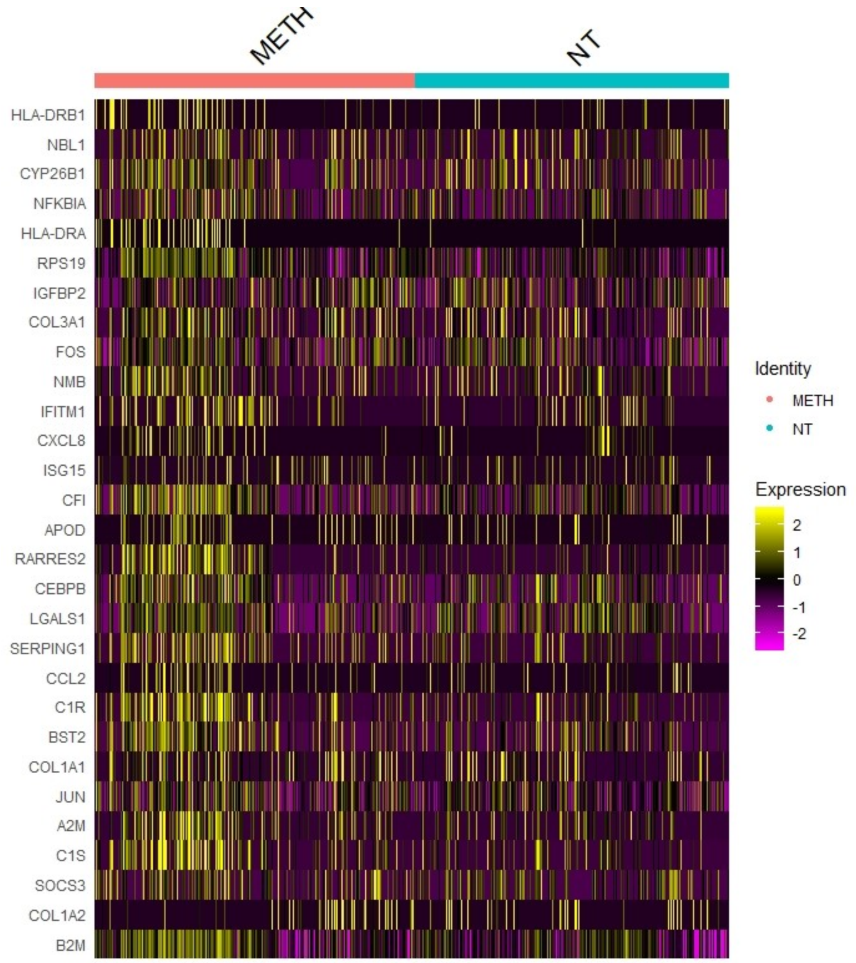

Supplementary Figure 4: Immune response genes upregulated as a result of METH treatment. Related to Figure 4.

(a) A dot plot of the genes from the set of genes upregulated by METH (Figure 2B) treatment identified by PANTHER to be related to immune response. Each cluster is divided by treatment, where blue dots represent cells from control organoid and red dots represent cells from METH treated organoids. Darker shades of blue and red and size of dots represent greater expression of genes and percentage of cells expressing the gene.

(b) A heatmap of the same genes from Figure 4A separated by treatment, for a holistic view of the data.

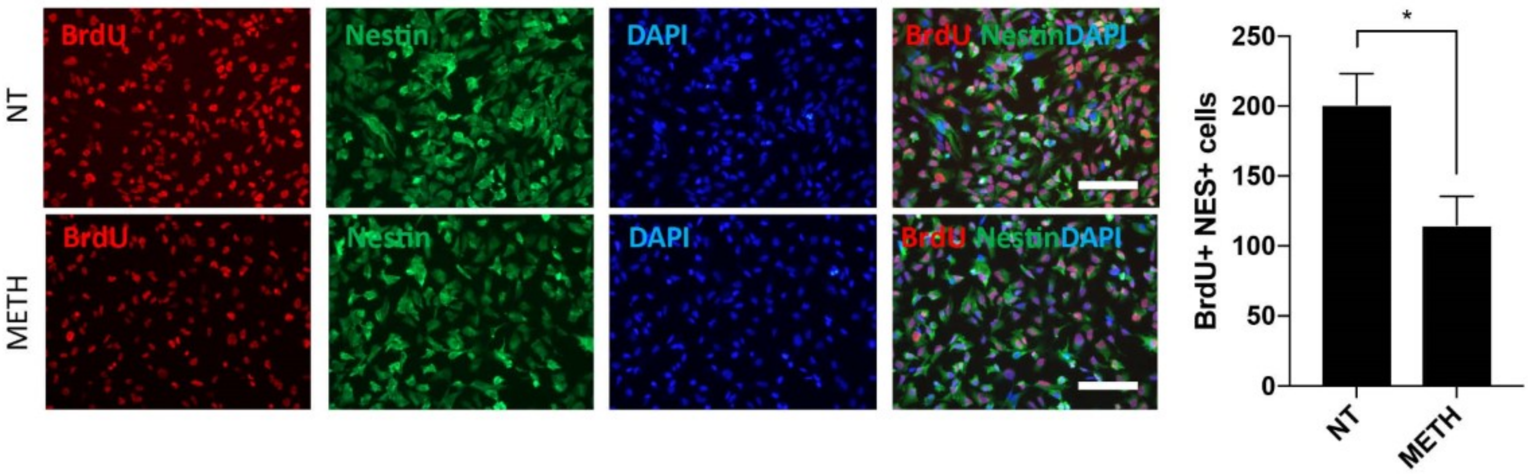

Supplementary Figure 5. METH treatment attenuates neural stem cell proliferation. Related to Figure 5.
Untreated (top row) and METH treated (bottom row) monolayer neural stem cells immunestained for BrdU (red), neural stem cell marker Nestin (green) and DAPI (blue) after 1 week of treatment with 5μM METH. Scale bar represents and 100μm.
